# Second Primary Cancers After Primary Breast Cancer Diagnosis in Israeli Women, 1992 to 2006

**DOI:** 10.1200/JGO.2016.003699

**Published:** 2016-06-29

**Authors:** Barbara G. Silverman, Irena Lipshitz, Lital Keinan-Boker

**Affiliations:** **Barbara G. Silverman**, **Irena Lipshitz**, and **Lital Keinan-Boker**, Israel Ministry of Health, Ramat Gan; **Barbara G. Silverman**, Tel Aviv University, Tel Aviv; and **Lital Keinan-Boker**, University of Haifa, Haifa, Israel.

## Abstract

**Purpose:**

Improvements in early detection and treatment have resulted in improved long-term survival from breast cancer, which increases the likelihood of the occurrence of second primary cancers. We calculated the risk of second primary cancers among Israeli women receiving a first primary breast cancer diagnosis.

**Methods:**

By using data from the Israel National Cancer Registry, we identified 46,090 women with invasive breast cancer diagnosed between 1990 and 2006 and non–breast primary cancers diagnosed subsequent to breast cancer diagnosis. We used life table analysis to calculate the risk of a second primary cancer and calculated standardized incidence ratios (SIRs) by using age-specific cancer risk in the general population of Israeli women as the standard and stratifying by diagnosis period (1992 to 1996, 1997 to 2001, 2002 to 2006) and age at diagnosis (< 50 and ≥ 50 years).

**Results:**

The probability of a second malignancy was 3.6% within 5 years, 8.2% within 10 years, and 13.9% within 15 years. The SIR for any second non–breast primary cancer was 1.26 (95% CI, 1.23 to 1.30). Significantly increased risks of colorectal, uterine, lung, ovarian, and thyroid cancer and leukemia were observed for the full follow-up period, which persisted after excluding the first 6 months after index diagnosis, although increased leukemia and colorectal cancer risks were no longer statistically significant. Women younger than age 50 years at initial diagnosis had a greater excess risk than women age 50 years and older (SIR, 1.77 [95% CI, 1.63 to 1.91] and 1.20 [95% CI, 1.15 to 1.24], respectively).

**Conclusion:**

The findings likely reflect a combination of personal risk factors (genetics, hormonal therapy, environmental exposures) as well as the effects of the initial cancer treatment and are unlikely to be explained by enhanced surveillance alone.

## INTRODUCTION

Breast cancer is the most frequently diagnosed cancer in Israeli women and constitutes approximately one third of all newly diagnosed tumors in both Jewish and Arab women every year. Improvements in early detection and treatment have resulted in increased long-term survival of breast cancer, which increases the likelihood of the occurrence of second primary cancers. Estimates from population-based studies of the increased risk of a second non–breast primary cancer after a breast cancer diagnosis range from 1% to 40%.^1-14^ Primary cancer diagnoses for which a significantly increased risk has been observed in women with a history of breast cancer include endometrial,^1,2,6,9,11-14^ ovarian,^6,9,11-13^ and colon^4-6,14^ as well as sites associated with previous radiotherapy.^4,9^ In most studies, women given a diagnosis of first primary breast cancer before the age of 50 years had a greater excess risk of a second primary cancer than those given a diagnosis at age 50 and older.^1-6,8,11,13,14^ The identification of factors associated with subsequent cancer diagnoses in women with breast cancer can guide long-term follow-up and screening of these patients. The aim of the current study was to characterize the factors associated with subsequent diagnoses of any type of cancer in a cohort of Israeli women who received a diagnosis of breast cancer from 1992 to 2006.

## METHODS

The Israel National Cancer Registry (INCR) was founded in 1960. Cancer reporting by hospitals, pathology and cytology laboratories, and other health care providers has been mandatory since 1982. The INCR covers the entire Israeli population (approximately 8 million), of which the ethnic distribution is as follows: 75% Jewish, 20% Arab, and 5% other ethnic groups. The following groups of diagnoses are recorded in the registry: all malignant neoplasms, excluding basal cell and squamous cell carcinoma of the skin; carcinoma in situ/high-grade (grade 3) intraepithelial neoplasias; and benign neoplasms of the brain and nervous system

The registry currently includes information on approximately 800,000 people; 30,000 new cases are entered per year. Sources of information include pathology reports, hospital discharge summaries, death certificates, and patient listings from cancer centers. Registry staff review all documents submitted to the registry and assign site and morphology codes according to the International Classification of Diseases for Oncology, Third Edition.^15^ Completeness of ascertainment has been estimated at 94% for solid tumors.^16^ Stage of disease at the time of diagnosis is determined on the basis of criteria established by the Middle East Cancer Consortium, of which INCR is a member.^17^ Middle East Cancer Consortium staging is primarily based on the criteria of the SEER Summary Staging Manual–2000.^18^ Demographic data and information on vital status are derived from the Central Population Registry of the Ministry of the Interior and updated at least annually. For purposes of disease surveillance, the Israeli population is divided, on the basis of data from the Ministry of Interior, into three ethnic subpopulations: Jewish, Arab, and other. Because previous work has shown that age-standardized cancer incidence in the Arab subpopulation is considerably lower than that in the Jewish or other subpopulations, we aggregated the Jewish and other subjects into a single group for the purpose of analysis.

The study cohort consisted of women who met the following criteria: diagnosed with invasive cancer of the breast (International Classification of Diseases for Oncology topography codes 50.0 to 50.9) between 1990 and 2006, excluding breast lymphomas; and no cancer diagnosis recorded before the date of breast cancer diagnosis

For all the women in the study cohort, we identified all cancers diagnosed at another site after the date of the first breast cancer diagnosis. We did not consider subsequent breast cancer diagnoses as second primary cancers for the purpose of this study. We considered two follow-up periods. The first period was calculated from the date of breast cancer diagnosis through the end of the observation period (defined as the earliest of the following: date of the first subsequent non–breast cancer diagnosis; date of death; or December 31, 2011). The second follow-up time was calculated from 6 months after the initial diagnosis through the end of the observation period. Subjects with more than one cancer during the follow-up period were censored after the first subsequent cancer diagnosis. To allow for examination of the effects of changes in treatment strategies over time, we divided the cohort into three groups according to year of diagnosis: 1992 to 1996, 1997 to 2001, and 2002 to 2006.

Crude incidence of subsequent cancers (overall and for selected sites) was calculated as the number of cases per 1,000 person-years of follow-up from time 0 (diagnosis of first primary invasive breast cancer) and from 6 months after time 0. Cumulative rates of second primary cancers within 5 years of breast cancer diagnosis, by period of diagnosis and age at diagnosis, were calculated by life table analysis. Expected numbers of cancer cases (overall and for selected sites) were calculated by using age-specific incidence data for Jewish Israeli women derived from the INCR database. Standardized incidence ratios (SIRs) were calculated as the ratio of observed to expected cases for several follow-up times (1, 5, 10, and 15 years). We based the expected number of cases on the rates in the Jewish population for two reasons: The majority of the women in the study population were Jewish, and the cancer rates are considerably higher in Jewish than in Arab women. Therefore, use of these rates resulted in a more-conservative estimate of the risk of second primary cancers in a population of women with breast cancer. Ninety-five percent CIs were calculated for SIRs that assumed a Poisson distribution. All data analyses were performed with SAS version 9.12 software (SAS Institute, Cary, NC).

## RESULTS

A total of 46,090 Israeli women with no previous cancer history were given a diagnosis of invasive breast cancer between 1990 and 2006 (42,355 Jewish; 2,296 Arab; 1,439 other ethnicity). Overall, 92% of cases were diagnosed in Jewish women, although the proportion of cases occurring in women in the Arab and other population groups increased with year of diagnosis (Table 1).

**Table 1:**
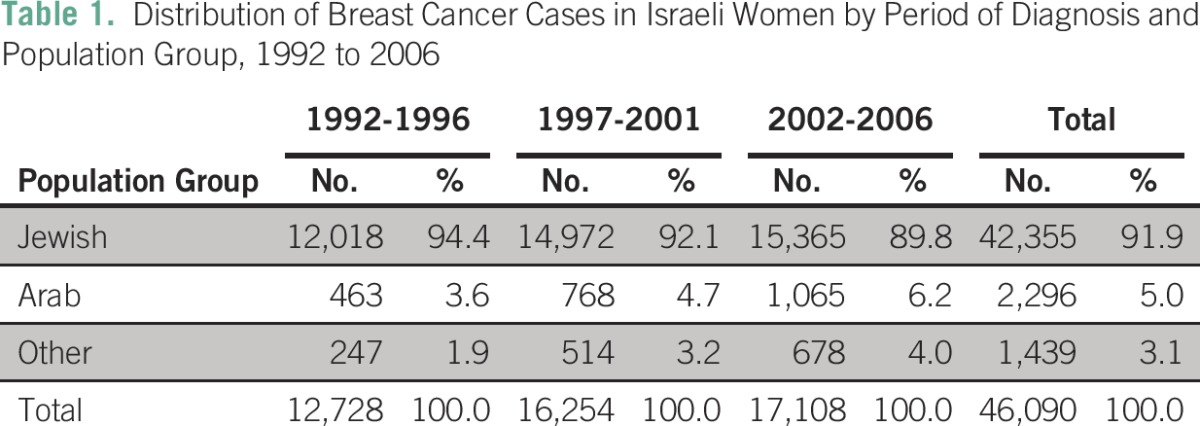
Distribution of Breast Cancer Cases in Israeli Women by Period of Diagnosis and Population Group, 1992 to 2006

Of women with breast cancer between 1992 and 2006, 3,980 (8.6%) were given a diagnosis of a second non–breast primary cancer during the follow-up period. Ninety-five percent of these cases (3,773) were among Jewish women, 3% among Arab women, and 2% among women of other ethnic backgrounds (Table 2). When the first 6 months after breast cancer diagnosis were excluded from the follow-up period, 3,619 second non–breast primary cancers were identified, with a distribution of ethnic backgrounds almost identical to that of cases identified during the full follow-up period. Twenty-five percent of index cases were diagnosed in women younger than age 50 years; of these women, 5.7% experienced a second non–breast primary cancer compared with 12.1% of those age 50 years and older at the time of the index diagnosis. Crude incidence of second non–breast primary cancers in this population was 10.6 per 1,000 person-years for the full follow-up period and 10.3 per 1,000 person-years for the shortened follow-up period.

**Table 2:**
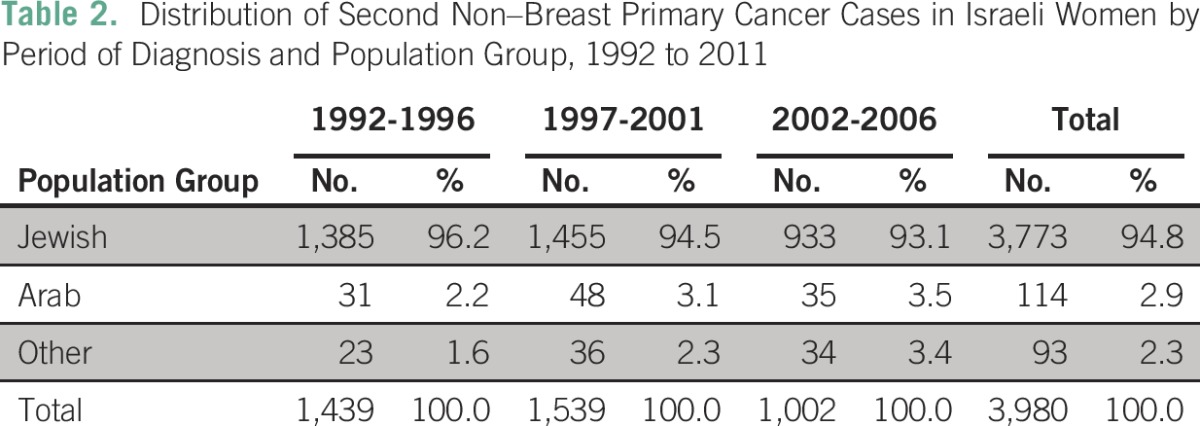
Distribution of Second Non–Breast Primary Cancer Cases in Israeli Women by Period of Diagnosis and Population Group, 1992 to 2011

The relatively small number of second primary cancers in the Arab population precluded stratification by age-group and cancer type. Therefore, all subsequent analyses, including calculation of SIR overall and for specific cancer types, focused on the Jewish and other subpopulations (43,794 women; 3,866 second non–breast primary cancers). This group contributed 363,333 person-years of follow-up beginning on the date of breast cancer diagnosis and 343,462 person-years when follow-up was assumed to begin 6 months after the breast cancer diagnosis. Mean available follow-up time per patient was 8.3 years (1992 to 1996, 10.6 years; 1997 to 2001, 8.9 years; 2002 to 2006, 6.1 years).

The most commonly diagnosed second primary cancers were colorectal, uterine, lung, ovarian, non-Hodgkin lymphoma, brain, malignant melanoma, thyroid, and leukemia (Table 3). During the more-conservative follow-up period, the most commonly occurring cancer types were consistent with those observed for the longer follow-up period but at slightly lower crude rates.

**Table 3:**
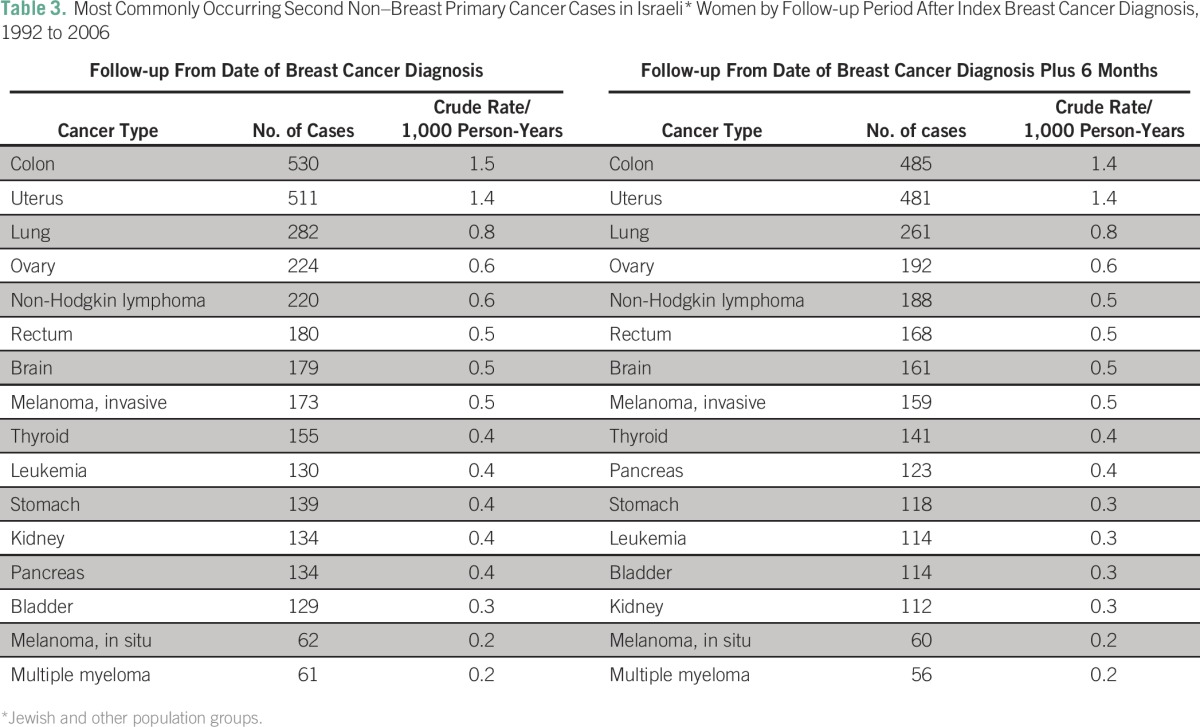
Most Commonly Occurring Second Non–Breast Primary Cancer Cases in Israeli* Women by Follow-up Period After Index Breast Cancer Diagnosis, 1992 to 2006

The SIR for a second non–breast primary cancer in women with a previous breast cancer diagnosis was 1.26 (95% CI, 1.23 to 1.30). The corresponding SIR for follow-up that commenced at 6 months after breast cancer diagnosis was 1.21 (95% CI, 1.16 to 1.25). Significantly increased risks of colorectal, uterine, lung, ovarian, and thyroid cancer and leukemia were observed for the full follow-up period. When follow-up excluded the first 6 months after breast cancer diagnosis, the increased risk of these cancers persisted, although for colorectal cancer and leukemia, this finding was no longer statistically significant (Table 4).

**Table 4:**
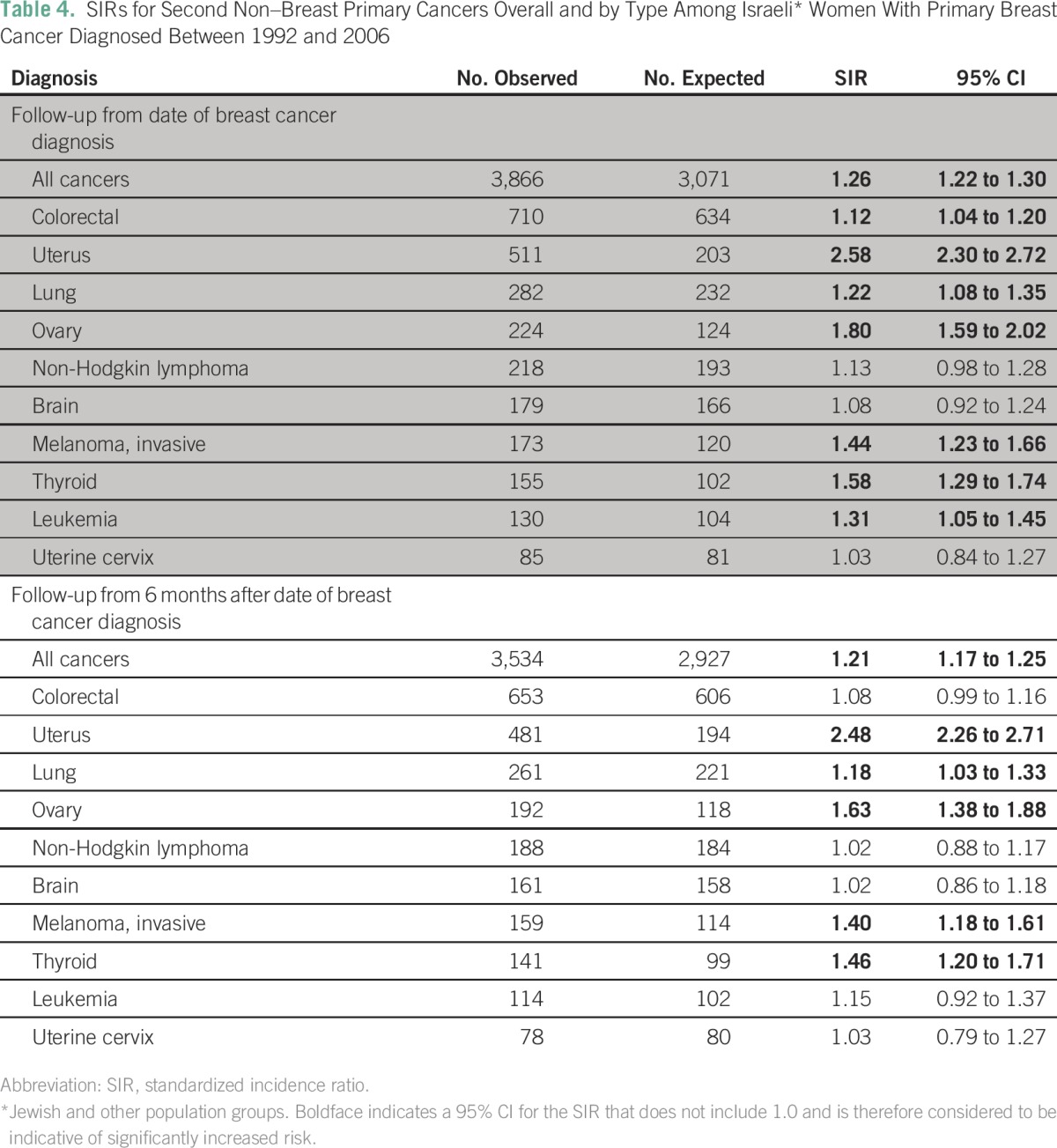
SIRs for Second Non–Breast Primary Cancers Overall and by Type Among Israeli* Women With Primary Breast Cancer Diagnosed Between 1992 and 2006

Stratification by age at diagnosis demonstrated greater excess risk for a second cancer among women given a diagnosis of breast cancer before age 50 years than in those age 50 years and older. Both groups, however, had a risk that was significantly higher than that for the general population of women the same age (SIR, 1.77 [95% CI, 1.63 to 1.91] and 1.20 [95% CI, 1.15 to 1.24], respectively).

Life table analysis indicated a cumulative probability of a second malignancy of 4.4% within 5 years, 9.3% within 10 years, and 15.3% within 15 years of the first breast cancer diagnosis. Probability of a second malignancy within 5 years of initial diagnosis did not vary significantly with period of first breast cancer diagnosis (1992 to 1996, 1997 to 2001, or 2002 to 2006); the number of patients remaining for analysis at 10 years for the group diagnosed between 2002 and 2006 was insufficient to allow for a comparison among all periods (Table 5). Women who were younger than 50 years of age at the time of index breast cancer diagnosis were less likely to receive a diagnosis of a second cancer within 5 years of follow-up than women age 50 and older at the time of index breast cancer diagnosis. The number of patients younger than age 50 years at that time of diagnosis who remained for analysis at 10 years was insufficient to allow for between-age comparison at that point in follow-up (Table 6).

**Table 5:**
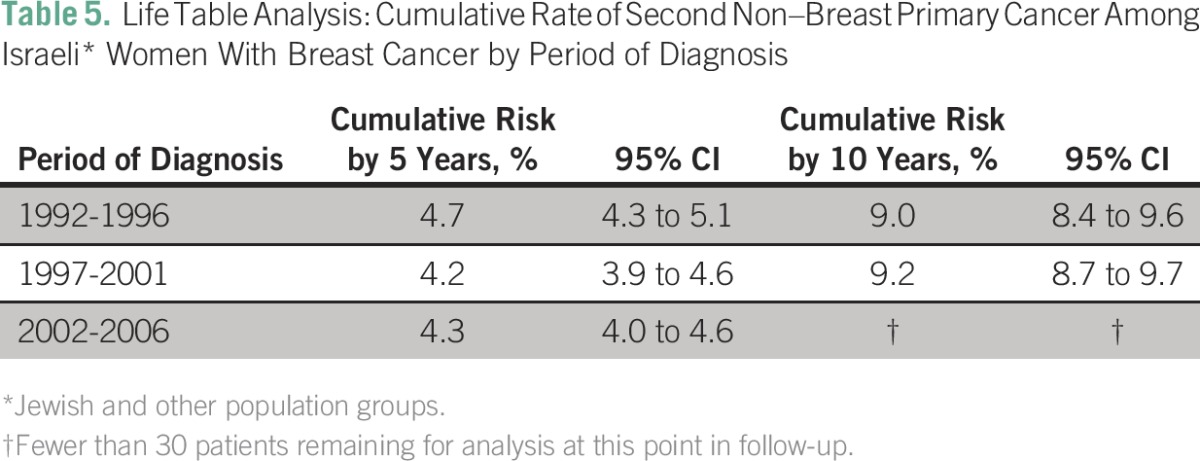
Life Table Analysis: Cumulative Rate of Second Non–Breast Primary Cancer Among Israeli* Women With Breast Cancer by Period of Diagnosis

**Table 6:**
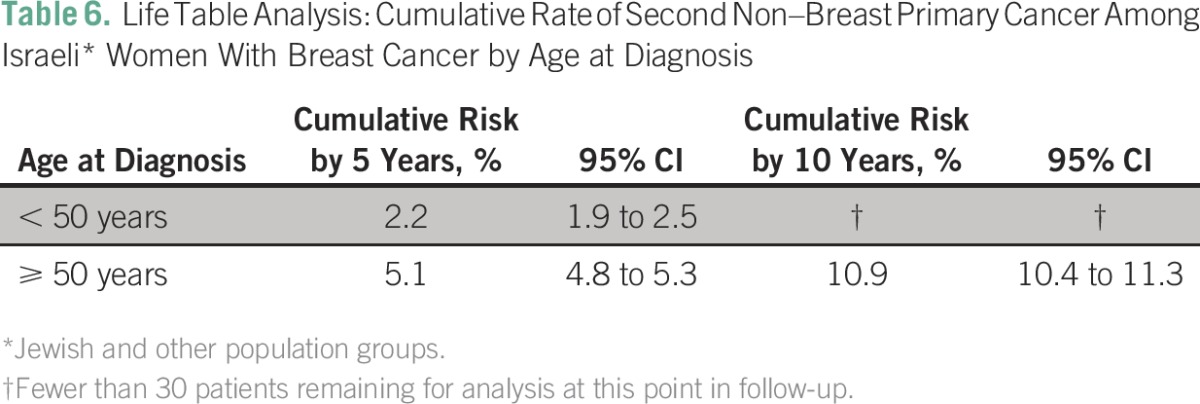
Life Table Analysis: Cumulative Rate of Second Non–Breast Primary Cancer Among Israeli* Women With Breast Cancer by Age at Diagnosis

## DISCUSSION

In a cohort of women with a first primary breast cancer diagnosed between 1992 and 2006, we observed a 25% increased risk of second non–breast cancers during follow-up compared with the risk in the general population, adjusted for age. Overall, the cumulative risk of a second non–breast primary cancer was 4.4% after 5 years of follow-up and 9.3% after 10 years.

A recent meta-analysis of 15 population- and hospital-based studies from Europe, Asia, and North America resulted in a pooled estimate of the increased risk of second cancers among patients with breast cancer of 1.17 (95% CI, 1.10 to 1.25) and found that the excess risk of a second primary cancer after breast cancer decreases with age at diagnosis of the index cancer (women age < 50 years: SIR, 1.51; 95% CI, 1.35 to 1.70; women age ≥ 50 years: SIR, 1.11; 95% CI, 1.02 to 1.21).^19^ Our estimate of the overall risk of a second cancer among patients with breast cancer is consistent with that analysis. As in previous studies, we found that although all women with breast cancer experience an increased risk of second primary cancer, younger women have a greater excess risk for a second primary cancer than the general population of women of the same age. As has been reported in other studies,^1-14^ the most pronounced excess risks in the current study population were for cancers of the uterus, ovary, and thyroid.

Several potential explanations exist for an increased risk of subsequent cancers in breast cancer survivors, including the effects of radiotherapy and hormone therapy, environmental factors, health behaviors, and genetics.^2^ Hormonal and radiation treatment of cancer carry an increased risk for the development of cancer. Duration of use of tamoxifen (but not the daily dose) has been associated with an increased risk of uterine cancer.^2,20^ Cancers most likely to be related to radiotherapy for breast cancer include leukemia and cancers of organs close to the breast, such as esophagus, lung and pleura, thyroid gland, stomach, and soft tissue sarcomas of thorax and upper limb.^2,3^

Women treated with radiation after mastectomy have been shown to have an increased risk for lung cancer, although radiation after lumpectomy does not carry this risk.^21^ Both radiation treatment and chemotherapy are associated with an increased risk of leukemia in patients with breast cancer^22^; the risk of leukemia in this population has been shown to increase with the intensity of treatment.^23^ SEER data indicate that the increased risk of a subsequent cancer associated with chemotherapy and radiation therapy is most pronounced in children and young adults and is not seen in older adults.^6^ This finding is consistent with the bulk of the literature that indicates that the excess risk of second primary cancers after breast cancer is most pronounced among younger women.

Another possible explanation for the increase in diagnoses of subsequent cancers in women with a history of breast cancer is that these women are under more intensive surveillance after treatment and therefore have a higher likelihood of subclinical lesions being detected. Sadetzki et al^24^ reported an increased risk of thyroid cancers in Israeli women previously treated for breast cancer and concluded that enhanced surveillance, common risk factors, and genetic predisposition were the likely causes for this finding. Mellemkjær et al^3^ reached a similar conclusion that although patients with breast cancer had an increased risk for subsequent thyroid cancer, this risk did not increase with latency and was accompanied by an increased risk of breast cancer occurring after thyroid cancer. Van Fossen et al^25^ noted a bidirectional association between breast and thyroid cancer, which suggests the existence of common risk factors for the two illnesses. We found that the exclusion of the first 6 months after breast cancer diagnosis from follow-up had little effect on the risk of subsequent thyroid cancer, which suggests that an increase in diagnoses of existing thyroid tumors at the time of breast cancer diagnosis is insufficient to explain the increased risk observed.

We used data from a large population-based national cancer registry to estimate the risk of second primary cancers a cohort of 46,090 Israeli women with a first diagnosis of breast cancer. The use of data from a well-established cancer registry for observational epidemiology research offers certain strengths. The INCR receives reports of cancer cases from all Israeli hospitals and pathology laboratories, thus the likelihood that cases of breast cancer and subsequent cancers diagnosed during the period of the study were excluded from the study cohort is minimal. All cases are reported by using a unique national identifier that allows for elimination of duplicate cases reported to the registry from different facilities. We focused on cases diagnosed between 1992 and 2006 to allow for an average follow-up time of 8.3 years. Population data available from the Israel Bureau of Statistics allowed us to calculated expected cancer rates in the general population for the purpose of calculating SIRs. Vital status information in the registry is supplemented with Israel Bureau of Statistics data to allow the censoring of patients who died during the follow-up period.

The INCR receives a limited amount of clinical data for patients with cancer. For this reason, we were unable to study the association of various types of breast cancer treatment on the occurrence of second cancers. No information on family history, personal risk factors (obesity, diet, or other health behaviors), or genetic testing is available for the patients in the cancer registry; therefore, these factors could not be taken into consideration in estimating second cancer risk. Genetic predisposition to cancer is an important consideration in the Israeli population. Between 2.0% and 2.5% of Ashkenazi Jewish women carry one or more of the founding mutations in the *BRCA1* and *BRCA2* genes.^26^ The cumulative risk at age 70 years of breast cancer in Ashkenazi women who are carriers of the *BRCA1* and *BRCA2* mutations has been estimated at 46% and 26%, respectively.^27^ Because mutations of the *BRCA1* or *BRCA2* genes contribute to an increased risk for cancers in other sites, such as the ovaries, cervix, uterus, pancreas, and colon, their high prevalence in the Israeli female population may limit the generalizability of the current data to populations with a low prevalence of *BRCA1/2* mutations.

Over the past 20 years, survival after breast cancer diagnosis has improved throughout the developed world, including Israel.^28^ With prolonged survival, however, comes an increased likelihood that patients treated for breast cancer in the past will be receive a diagnosis of additional primary cancers as a result of underlying genetic and other risk factors related to the primary breast cancer and to treatment of the original illness. Breast cancer survivors tend to be more intensive users of medical services than other women their age.^29^ Specialist and primary care providers must take advantage of this continued contact to educate patients about their risk for second cancers and implement appropriate preventive and screening procedures tailored to their patients’ individual risks.
